# Vitamin D and the Risk of Atrial Fibrillation - The Rotterdam Study

**DOI:** 10.1371/journal.pone.0125161

**Published:** 2015-05-01

**Authors:** Anna Vitezova, Natasha S. Cartolano, Jan Heeringa, M. Carola Zillikens, Albert Hofman, Oscar H. Franco, Jessica C. Kiefte-de Jong

**Affiliations:** 1 Department of Epidemiology, Erasmus MC University Medical Center, Rotterdam, The Netherlands; 2 Department of Internal Medicine, Erasmus MC University Medical Center, Rotterdam, The Netherlands; University of Alabama at Birmingham, UNITED STATES

## Abstract

Atrial fibrillation (AF) is the most common chronic arrhythmia and it increases the risk of cardiovascular morbidity and mortality. Still there is not a complete understanding of its etiology and underlying pathways. Vitamin D might regulate renin-angiotensin-aldosterone system and might be involved in inflammation, both implicated in the pathophysiology of AF. The objective of this work was to investigate the association between vitamin D status with the risk of AF in the elderly. This study was conducted within the Rotterdam Study, a community-based cohort of middle-aged and elderly participants in Rotterdam, The Netherlands. We had 3,395 participants who were free of AF diagnosis at the start of our study and who had vitamin D data available. We analyzed the association between serum 25-hydroxivitamin D (25(OH)D) and incidence of AF using Cox regression models. Vitamin D deficiency was defined as serum 25(OH)D concentrations <50nmol/l, insufficiency between 50nmol/l and 75nmol/l, while serum 25(OH)D concentrations equal to and above 75nmol/l were considered as adequate. After mean follow-up of 12.0 years 263 (7.7%) participants were diagnosed with incident AF. Vitamin D status was not associated with AF in any of the 3 multivariate models tested (model adjusted for socio-demographic factors and life-style factors: HR per 10 unit increment in serum 25(OH)D 0.96, 95% CI: 0.91-1.02; HR for insufficiency: 0.82, 95%CI: 0.60-1.11,and HR for adequate status: 0.76, 95%CI: 0.52-1.12 compared to deficiency). This prospective cohort study does not support the hypothesis that vitamin D status is associated with AF.

## Introduction

Atrial fibrillation (AF) is the most common chronic arrhythmia and it has a significant effect on morbidity and mortality [1–4]. Since AF is mainly a disease of the elderly, the prevalence of this arrhythmia is expected to increase due to aging populations [5], which has a major impact on healthcare expenditure [6, 7].

Despite extensive research on AF, there is still not a complete understanding of its etiology and underlying pathways. Risk factors for AF include older age, male sex, hypertension, valvular heart disease, congestive heart failure, ischemic heart disease and hyperthyroidism [8–10].

Vitamin D is associated with calcium metabolism and bone health. However, vitamin D receptors (VDRs) have been found in cells throughout the body, such as cardiomyocytes and endothelial cells [11], suggesting this hormone has additional functions in the human body. Furthermore, vitamin D deficiency is highly prevalent in Western populations and has been considered a global health issue, especially in the elderly [12–17].

Amongst different mechanisms through which vitamin D is involved in human health, vitamin D can regulate the renin-angiotensin-aldosterone system (RAAS) activity and has also been involved in the inflammatory processes, both implicated in the pathophysiology of AF, therefore suggesting a potential role of vitamin D in the etiology of AF. Nevertheless, only a few studies have analyzed the possible association between vitamin D deficiency and AF. Two cohorts investigating the association between vitamin D status and atrial fibrillation reported opposite results [18, 19]. Thus, it remains unknown whether there is an association between AF and vitamin D status.

The aim of our study was to investigate the association between serum levels of 25-hydroxyvitamin D (25(OH)D) with the risk of AF using data from a community-based cohort study of middle aged and elderly participants.

## Methods

### Study Design

This study was conducted among individuals from The Rotterdam Study, a population based prospective cohort study investigating frequency and determinants of disease in the middle aged and the elderly. The Rotterdam Study started in 1990 when 10,275 inhabitants of the Ommoord district of Rotterdam, The Netherlands, aged 55 years and older, were invited to participate in the study. Of these 7,983 (78%) provided written consent to participate. They were interviewed at home and subsequently examined in the research center from 1990 to 1993. The examinations were repeated every 3–4 years. For this study, the third examination round (1997–1999) was considered as baseline when 25-hydroxivitamin D (25(OH)D) levels were assessed. All participants gave informed consent and the study was approved by the medical ethics committee of the Erasmus Medical Center, Rotterdam. The study is described in more details elsewhere [20].

### Study Population

From 7,983 participants enrolled to the first examination round of The Rotterdam Study, 3,828 had data on serum 25(OH)D available at the examination round between 1997–1999. Of these, 434 participants were excluded because they had no data on AF, had prevalent AF or did not have follow-up information recorded. The remaining study population consisted of 3,395 participants of The Rotterdam Study.

### Assessment of 25(OH)D

Serum 25(OH)D concentrations were assessed in 3,828 participants of the Rotterdam Study. The measurements were performed with a electrochemiluminescence immunoassay (COBAS, Roche Diagnostics GmbH, Germany). Test sensitivity was 10nmol/L, serum 25(OH)D concentrations ranged from 7.5nmol/L to 175nmol/L, the within-run accuracy was less than 7.8% and intermediate precision accuracy was less than 13.1%. Serum 25(OH)D concentrations were analyzed both as a continuous variable and as a categorical variable according to cut-offs proposed by M. Holick [21]. Participants were categorized as being vitamin D deficient (<50 nmol/L), insufficient (50–75 nmol/L) or having adequate vitamin D status (≥75 nmol/L) according to their serum 25(OH)D concentrations.

### Assessment of Atrial Fibrillation

Between 1997–1999, AF was collected using 10-second, 12-lead electrocardiography (ECGs) recorded with an ACTA electrocardiograph (ESaOte, Florence, Italy) and by screening of general practitioners (GPs) records from Ommoord region. During follow-up, ECGs were performed during the re-examinations every 3–4 years. In addition, GPs weekly updated information on AF based on their own records and hospital discharge letters. Information on hospital discharge was also collected from a national registration system (Landelijke Medische Registratie system). A diagnosis of AF was only accepted when it was supported by a ECG diagnosis. The ECGs done at baseline and during follow-up were stored digitally, and analyzed by the modular ECG analysis system (MEANS), which has high specificity (99.5%) and high sensibility (96.6%) for detection of arrhythmias [22, 23].

Two research physicians, who were blinded for the MEANS result, verified all ECGs with a diagnosis of atrial fibrillation, atrial flutter or any other arrhythmia done by the computer system [10]. In case of disagreement, diagnosis of a cardiologist was considered as decisive. In this study no distinction was made between atrial fibrillation and atrial flutter [24, 25]. A participant was not considered to have AF if a transient AF occurred during myocardial infarction or during cardiac operative procedures. If AF occurred during the process of dying and was not the cause of death, the person was not considered as a case and was censored on the date of AF diagnosis.

### Assessment of Confounders

Socio-demographic, lifestyle and medical information was assessed during a home interview. Education was assessed using the highest level attained. Low education level was considered as primary education only or primary education with uncompleted higher education. Income was assessed as net income per year. Income was categorized as high or low income, low income was considered as less than 35.999 euros per year and high income above or equal to 35.999 euros per year. Smoking was assessed by using questions on current and past smoking of cigarettes, cigars, or pipe. The information on medication use included information on lipid lowering and blood pressure lowering drugs. Information on diet were obtained through a 170-item validated semiquantitative food frequency questionnaire (SFFQ)[26]. From that an overall healthy diet score representing adherence to the Dutch dietary guidelines was calculated as described previously by van Lee et al [27]. Physical activity was assessed using a validated adapted version of Zutphen Physical Activity Questionnaire[28]. Questions on housekeeping activities were added to the original questionnaire that already included questions on walking, cycling, gardening, hobbies, and diverse sports.

During the examination visit (1997–1999) physiological measurements were performed as well as blood collection. Blood pressure (BP) was measured twice on the right arm in the sitting position with a random zero sphygmomanometer. The average of two consecutive measurements was used. Body mass index (BMI) was calculated by dividing weight in kilograms by height in meters squared. Waist circumference was measured at the level midway between the lower rib margin and the iliac crest with the participant in a standing position. Diabetes mellitus was considered to be present if fasting (8–14 h) glucose value was 7.0 mmol/l or higher or a random or postload glucose value was 11.1 mmol/l or higher in any of the examination rounds up to 1997–1999 or if a participant used anti-diabetic medication or diet treatment and was registered by a general practitioner as having diabetes. The estimated glomerular filtration rate (eGFR) was computed according to the simplified Modification of Diet in Renal Disease (MDRD) formula [29], chronic kidney disease was defined as a eGFR below 60 mL/min/1.73 m^2^. A history of stroke at study entrance was defined as a self-reported stroke that was confirmed by medical records. Subsequently up to 1997–1999 stroke was assessed from the follow up information obtained from the GP’s files. At study entrance history of myocardial infarction (MI) was positive if MI was reported during baseline interview and confirmed by hospital admission and/or MI was present on the ECG. During follow up until 1997–1999 MI was confirmed using medical records [30]. At the entrance to the Rotterdam Study cases of heart failure were classified according to definition by the European Society of Cardiology based on following: 1) at least two symptoms of heart failure present (shortness of breath, swelling of ankles, or pulmonary crepitation), 2) use of medication prescribed for heart failure (diuretics, glycosides, or ACE inhibitors) together with cardiovascular disease [31–33]. During follow up until 1997–1999 the information on heart failure was obtained from general practitioner’s records [33]. To identify patients potentially eligible for use of vitamin D medication we used diagnosis of osteoporosis assessed by dual-energy x-ray absorptiometry as a proxy.

### Statistical analyses

Cox proportional hazards regression was used to relate serum 25(OH)D concentrations with incident atrial fibrillation. Proportional hazard assumption was tested including time variable x serum 25(OH)D and time variable x cut-offs of serum 25(OH)D into the model. Follow-up duration in years was used as time variable. The participants were followed from date of the third visit to the research center of the Rotterdam Study (1997–1999) to the end of follow up (March 22, 2010). Subjects were censored when they died or were lost to follow up. Prevalent cases of atrial fibrillation between study entrance and 1999 were excluded from the analyses. Multiple imputation procedure was performed to reduce bias from missing data (0–22%). Ten imputations were done using Markov chain Monte Carlo method (S1 Table). Potential confounders were chosen based on change in the effect estimates and/or based on the literature [34–36]. Three multivariate models were created for Cox regression analyses. The first model was the crude model, adjusted for age and gender. The second model was adjusted additionally for the following confounders: net household income, highest education level attended, BMI, physical activity, diet quality score, current smoking, and season and year when the blood was drawn. The third model was additionally adjusted for potential mediators: use of serum lipid lowering drugs, use of BP lowering drugs, systolic BP, and prevalent diseases (cardiovascular diseases including coronary heart disease, heart failure, and stroke; chronic kidney disease; diabetes mellitus). Stratification was performed by age, gender, smoking, and use of BP lowering drugs. Also, interaction terms of covariate x serum 25(OH)D and covariate x dummy variables of serum 25(OH)D cut-offs were created for assessing possible effect modification by these variables. Additional analyses included testing for potential confounding or modification by serum calcium; firstly the models were adjusted for serum calcium concentrations; secondly, the interaction between serum calcium and serum 25(OH)D was tested; thirdly the analyses were performed excluding participants with hypercalcaemia. Sensitivity analyses were performed by censoring cases of coronary heart disease (CHD) that occurred before the onset of AF and excluding participants potentially eligible for receiving vitamin D supplementation (i.e. subjects with osteoporosis). Data analyses were performed using SPSS version 21.0 (SPSS IBM, New York, USA).

## Results

Baseline characteristics of the study population are summarized in Table 1. The population for analysis consisted of 3,395 participants. After a mean follow-up of 12.0 years 263 (7.7%) participants were diagnosed with incident atrial fibrillation. The median (range) age of the study population was 71(44) years and 2,007 (59.1%) participants were female. The mean (SD) 25(OH)D concentration was 49.3 (25.4) nmol/l. According to cutoffs, 57% of the study population had deficiency (<50nmol/l), 27% had insufficiency (50-75nmol/l) and 16% had adequate vitamin D status (≥75nmol/l). Individuals with vitamin D deficiency or insufficiency were more often female, more likely to be older, to have lower education degree, lower income, higher BMI, and more often had diabetes mellitus and cardiovascular disease (CVD) (Table 1). The percentage of missing data varied from 0 percent for variables like age and gender up to 12.9 percent for data on physical activity (data not shown).

**Table 1:** Baseline characteristics of study population according to vitamin D status.

	Total	Deficient	Insufficient	Adequate
		<50nmol/l	50-75nmol/l	≥75nmol/l
Individuals N(%)	3395 (100)	1939 (57,1)	909 (2608)	547 (160,1)
Age (years)**	*71*,*0 (44*,*4)*	*72*,*8 (44*,*3)*	*69*,*3 (2902)*	*68*,*3 (27*,*1)*
Females N(%)	2007 (5901)	1314 (67,8)	464 (51)	229 (41,9)
Serum 25(OH)D (nmol/l)*	49,3 (25,4)	31,4 (10,7)	61,1 (7)	93,1 (15,4)
Follow up time (years)**	12 (16)	11 (16)	12 (16)	13 (16)
AF incidence N(%)	263 (7,7)	167 (8,6)	61 (6,7)	35 (6,4)
Low education N(%)#	1006 (29,6)	646 (33,3)	232 (25,5)	128 (23,4)
Diet quality score (DHDI)* #	48,7 (9,6)	48,9 (9,5)	48,8 (9,6)	47,9 (9,6)
Current smokers N(%)	557 (16,4)	333 (17,2)	128 (14,1)	86 (15,7)
BMI (kg/m2)*	26,9 (3,9)	27,2 (4,2)	26,4 (3,4)	26,3 (3,4)
Waist circumference (cm)*	93,4 (11,4)	93,9 (11,9)	92,7 (10,6)	92,8 (10,6)
Systolic BP (mmHg)*	143 (21)	145 (21)	142 (20)	141 (20)
Diastolic BP (mmHg)*	75 (11)	75 (11)	75 (11)	76 (10)
Use of BP lowering medication N(%)	940 (27,7)	600 (30,9)	227 (25)	113 (20,7)
Presence of DM N(%)	487 (1403)	334 (17,2)	105 (11,6)	48 (8,8)
Presence of CVD N(%)	495 (14,6)	315 (16,2)	116 (12,8)	64 (11,7)
Presence of CKD N(%)	479 (14,1)	302 (15,6)	101 (11,1)	77 (14,1)
Use of lipid lowering medication N(%)	512 (15,1)	305 (15,7)	127 (14)	80 (14,6)
Hypercalcaemia N(%)	61 (1,8)	44 (1,1)	11 (1,2)	6 (2,3)
Hypocalcaemia N(%)	21 (0,6)	18 (0,9)	2 (0,2)	1 (0,2)

BMI- body mass index; BP- blood pressure; DM- diabetes mellitus; CVD- cardiovascular disease (considered as the presence of coronary artery disease, heart failure or stroke); CKD- chronic kidney disease.

*Mean (Standard Deviation);

**Median (Range);

^#^ Data collected prior to serum 25(OH)D assessment;

Note: prevalent cases of AF were excluded from the analysis; Imputed data are shown.

After adjustment for age and gender no significant association was found between vitamin D status and atrial fibrillation, both when serum 25(OH)D was analyzed continuously (HR per 10 unit increment in serum 25(OH)D: 0.95, 95% CI: 0.90–1.01) or by cutoffs (HR for insufficient levels: 0.79, 95%CI: 0.58–1.07 and HR for adequate levels: 0.74, 95%CI: 0.50–1.08, compared to deficient levels). The results remained non-significant after further adjustments in model 2 (HR per 10 unit increment in serum 25(OH)D: 0.96, 95% CI: 0.91–1.02, HR for insufficient levels: 0.82, 95%CI: 0.60–1.11, HR for adequate levels: 0.76, 95%CI: 0.52–1.12) (Table 2). Additional adjustment for potential mediators did not change the results (Table 2).

**Table 2:** Serum 25(OH)D and incidence of atrial fibrillation.

	Model 1	Model 2	Model 3
	HR (95% CI)	HR (95% CI)	HR (95% CI)
**25(OH)D cutoffs**			
** Deficiency**	*Reference*	*Reference*	*Reference*
** Insufficiency**	0,79 (0,58, 1,07)	0,82 (0,60, 1,11)	0,85 (0,62, 1,15)
** Adequate**	0,74 (0,50, 1,08)	0,76 (0,52, 1,12)	0,81 (0,55, 1,20)
25(OH)D continuous*	0,96 (0,90, 1,01)	0,96 (0,91, 1,02)	0,97 (0,92, 1,03)

Model 1 adjusted for age and gender; Model 2 adjusted for age, gender, income, education, BMI, physical activity, diet quality score, smoking status and season and year when the blood was drawn; Model 3 adjusted for all covariates in model 2 plus use of serum lipid lowering medication, use of blood pressure lowering medication, systolic blood pressure and baseline diseases: cardiovascular diseases (coronary heart disease, heart failure, stroke), chronic kidney disease and diabetes mellitus;

*Results per 10 units of serum 25(OH)D (nmol/l); Prevalent cases of AF were excluded from the analysis; Imputed data are shown.

We tested the interaction of serum 25(OH)D with gender, age, current smoking and use of BP lowering drugs. None of the interaction terms entered in the model were statistically significant. We also performed stratifications based on these covariates, but no significant associations were seen in these strata (data not shown). Additional adjustment for serum calcium did not change the results (data not shown). We also tested for the interaction between serum calcium and serum 25(OH)D but this was also not significant (data not shown). Excluding participants with hypercalcaemia did not change the results significantly (data not shown).

Furthermore, we did sensitivity analyses. Firstly, we censored cases of CHD that occurred before the incidence of AF. Secondly, we excluded participants on therapy with vitamin D. Neither of the two sensitivity analyses markedly changed the results (data not shown).

## Discussion

Overall, vitamin D status was not associated with the incidence of AF in this prospective cohort study. Besides, no association was found after censoring of CHD cases that occurred before AF. Stratification according to potential effect modifiers also did not change the results.

Vitamin D has attracted much attention for its potential relation with non-skeletal disorders. Experimental research has supported the role of vitamin D deficiency in several cardiovascular diseases [37]. The role of vitamin D deficiency in the onset of AF was suggested because of numerous potential mechanisms described previously. Vitamin D regulates inflammatory responses and up-regulates the expression of anti-inflammatory cytokines as IL-10 according to in-vitro experiments [38]. Also, vitamin D regulates RAAS activity. Activated RAAS can lead to oxidative stress and inflammation both of which can culminate in AF [39, 40]. It is hypothesized that tissue angiotensin II can induce apoptosis of cardiomyocytes and this way can contribute to changes in atrial structure. [41]. Also, angiotensin II can modulate the expression of several ion channels in cardiomyocytes [42]. Another way how RAAS might be involved in pathophysiology of AF is the altered atrial expression of angiotensin receptors found in patients with AF [43]. Moreover, angiotensin antagonists prevent the electrical atrial remodeling seen in AF [44]. Finally, experiments with mice show higher serum angiotensin II and renin activity in mice knocked-out for VDR [45]. In addition, mice unable to synthesize 1,25(OH)_2_D due to 1-alpha—hydroxylase deficiency have elevated RAAS activity, hypertension and cardiac hypertrophy [46].

Until now not much research has been done on the association of vitamin D deficiency and the risk of AF. We identified two cohorts reporting on this topic. The first was Framingham Heart Study and analyses were conducted by Rienstra and colleagues [19]. The study population counted 2930 participants out of which 15 percent developed AF during follow-up. The mean age was 66 years. Authors report results similar to ours, HR per SD increment in 25(OH)D fully adjusted model: 0.99, 95% CI: 0.88–1.10 [19]. The second cohort coming from Kansas, USA was used to investigate the association between vitamin D deficiency, vitamin D supplementation and numerous outcomes, including AF [18]. This was a large cohort counting 10,899 participants with mean age 58 years. Only 5 percent of these participants developed AF. Even though the authors reported vitamin D deficiency significantly associated with several cardiovascular diseases and found vitamin D deficiency to be a strong predictor of all-cause mortality they also found vitamin D deficiency negatively associated with risk of AF (OR 0.83, 95% CI 0.693, 0.984). However, the authors obtained these results using univariate analysis. It might be very likely that these results were confounded and thus not reflecting the true nature of the relation between vitamin D status and AF. In their case analysis Qayyum and colleagues found no association between vitamin D status and AF regardless of valvular disease [47].

Finally, two cross-sectional studies reporting on vitamin D deficiency and AF [48, 49] found an inverse association between vitamin D status and AF not related to valvular heart disease. Valvular AF and non-valvular AF is an important classification from therapeutic perspective. There is not much literature supporting a difference in the mechanisms underlying these two conditions [50]. Unfortunately, we had no information on valvular heart disease to reproduce these latter findings. However, our study may mainly reflect the relation between vitamin D status and AF not related to valvular heart disease, since it has been estimated that only about 10% of the AF cases are due to valvular heart disease in The Netherlands [51].

In our study we decided to stratify analyses by gender, age, current smoking and use of BP lowering drugs because of the interaction they could play with the incidence of AF. Older age is a major risk factor for AF. In our cohort it was already shown that the incidence rate of AF increased from 1.1 cases at ages 55–60 to 20.7 cases at ages 80–85 per 1000 person-years [10]. Similar increases were reported in several studies. The aging process is related to changes in atrial structure that favor AF development [35, 52]. Men and women differ in incidence and prognostic related to AF [10, 53, 54]. The mechanisms behind these findings are not totally clear. However it was shown that men and women have a different expression of ion channels in atria and cardiac myocytes. Also, fibroblasts express functional estrogen receptors. Moreover, genes related to atrial remodeling can be regulated by estrogens [55–57]. We hypothesized that different mechanisms could be related to development of AF in men and women and vitamin D deficiency could interfere differently according to gender. Smoking was shown to alter the effect of vitamin D in specific diseases; low concentrations of vitamin D were a risk factor for tobacco related cancers [58]. In addition, smoking is a risk factor for development of AF in the Rotterdam Study [59]. Some classes of blood pressure lowering drugs were also shown to prevent atrial remodeling in animal models [60]. Although the findings in humans are controversial [60], we hypothesized that some classes of antihypertensive drugs could interfere with effects of vitamin D status on the atria. In our analyses, there was no differential association between vitamin D status and AF by strata of age, gender, smoking and hypertension, however our results may have been limited due to small sample size to detect any potential effect modification. Furthermore, we expected that CHD could be a potential mediator in the association between vitamin D status and incidence of AF since several studies has shown an association between vitamin D status and CHD [61] but also association between CHD and AF [62]. However, no changes were seen in the HRs when we censored participants with any CHD prior to AF.

The strengths of this study are the prospective cohort design and the extensive information on covariates. There are also some limitations that need to be addressed. Serum 25(OH)D was measured only once and may not reflect the values during the onset of AF. Also, the lack of data on valvular heart disease and parathyroid hormone levels limited replication of previous studies on this topic. We diagnosed AF with ECG or from medical records. However, many of the AF cases are asymptomatic, which may have underestimated the true prevalence of AF in our study population. However, this probably did not affect the direction of our results since this misclassification likely happened independently of vitamin D status. Furthermore, in our study the participants were Caucasians, therefore our findings must be interpreted with caution for other ethnic populations.

## Conclusion

In conclusion, our prospective cohort study does not support the hypothesis that vitamin D status may play a role in the etiology of AF in the elderly. Further studies using repeated measurements of serum 25(OH)D as well as performing analysis in other populations may shed further light on whether the role of vitamin D status in the etiology of AF is justified.

## Supporting Information

S1 Table (PDF)
