# Supplementary material for: Vitamin D and the Risk of Atrial Fibrillation - The Rotterdam Study
Source: PLoS One. 2015 May 1;10(5):e0125161. doi: 10.1371/journal.pone.0125161 (PMC4416919; doi:10.1371/journal.pone.0125161)
Supplement: S1 Table — (PDF) [file pone.0125161.s001.pdf]

S1 Table. Details of the multiple imputation modelling

	Multiple imputation procedure
Software used:	SPSS 21.0 for windows.
Imputation method and key settings:	Fully conditional specification (Markov chain Monte Carlo method); Maximum iterations: 10.
No of imputed data sets created:	10
Variable included in the imputation procedure and used in main analyses: (imputed and used as predictors of missing data)	Total energy intake, DHDI, alcohol intake, physical activity, smoking, income, education, weight, height, waist circumference, systolic and diastolic blood pressure, eGFR, serum levels of creatinine, TSH, triglycerides, total cholesterol, HDL cholesterol and glucose, prevalent cardiovascular diseases, antidiabetic therapy, serum lipid lowering drugs, thyroid therapy, blood pressure lowering drugs, family history of myocardial infarction, diabetes mellitus, dementia and stroke and season of blood draw.
Variables not used in main analyses or without missing data but used as predictors of missing data to increase plausibility of missing at random assumption:	Age, sex, vitamin D intake, serum levels of vitamin D, insulin, gamma glutamyl transpeptidase, total bilirubin, magnesium, sodium, calcium, potassium, phosphate and homocysteine and incidence of AF.
Treatment of non-normally distributed variables	Predictive mean matching
Treatment of binary/categorical variables	Logistic regression
